# Lipoteichoic Acid from *Lacticaseibacillus rhamnosus* GG as a Novel Intracanal Medicament Targeting *Enterococcus faecalis* Biofilm Formation

**DOI:** 10.1007/s12275-024-00165-6

**Published:** 2024-09-30

**Authors:** Ji-Young Yoon, Somin Park, Dongwook Lee, Ok-Jin Park, WooCheol Lee, Seung Hyun Han

**Affiliations:** 1 Department of Conservative Dentistry, and DRI, School of Dentistry, Seoul National University, Seoul, 03080 Republic of Korea; 2 Department of Conservative Dentistry, Seoul National University Bundang Hospital, Seongnam, 13620 Republic of Korea; 3 Department of Oral Microbiology and Immunology, and DRI, School of Dentistry, Seoul National University, Seoul, 08826 Republic of Korea

**Keywords:** Biofilm, *Enterococcus faecalis*, *Lactobacillaceae*, Lipoteichoic acid, Root canal medicaments

## Abstract

The demand for safe and effective endodontic medicaments to control *Enterococcus faecalis* biofilms, a contributor to apical periodontitis, is increasing. Recently, lipoteichoic acid (LTA) of family *Lactobacillaceae* has been shown to have anti-biofilm effects against various oral pathogens. Preliminary experiments showed that LTA purified from *Lacticaseibacillus rhamnosus* GG (Lgg.LTA) was the most effective against *E. faecalis* biofilms among LTAs from three *Lactobacillaceae* including *L. rhamnosus* GG*, Lacticaseibacillus casei,* and *Lactobacillus acidophilus*. Therefore, in this study, we investigated the potential of Lgg.LTA as an intracanal medicament in human root canals infected with *E. faecalis.* Twenty eight dentinal cylinders were prepared from extracted human teeth, where two-week-old *E. faecalis* biofilms were formed followed by intracanal treatment with sterile distilled water (SDW), *N*-2 methyl pyrrolidone (NMP), calcium hydroxide (CH), or Lgg.LTA. Bacteria and biofilms that formed in the root canals were analyzed by scanning electron microscopy and confocal laser scanning microscopy. The remaining *E. faecalis* cells in the root canals after intracanal medicament treatment were enumerated by culturing and counting. When applied to intracanal biofilms, Lgg.LTA effectively inhibited *E. faecalis* biofilm formation as much as CH, while SDW and NMP had little effect. Furthermore, Lgg.LTA reduced both live and dead bacteria within the dentinal tubules, indicating the possibility of minimal re-infection in the root canals. Collectively, intracanal application of Lgg.LTA effectively inhibited *E. faecalis* biofilm formation, implying that Lgg.LTA can be used as a novel endodontic medicament.

## Introduction

*Enterococcus faecalis,* a facultative anaerobic Gram-positive bacterium, is closely associated with persistent endodontic infections leading to apical periodontitis (Zhang et al., 2015). *E. faecalis* possesses various virulence factors for deep penetration into dentinal tubules, biofilm formation, and resistance to nutritional deprivation (Love, 2001; Rôças et al., 2004; Stuart et al., 2006). Under starvation conditions, *E. faecalis* becomes resistant to antimicrobial agents (Portenier et al., 2005). These factors pose significant challenges in eliminating *E. faecalis* from failed endodontic treatments.

Calcium hydroxide (CH), which is a widely-used intracanal medicament, possesses antibacterial effects due to its strong alkalinity (Estrela et al., 1999). However, *E. faecalis* can resist CH by the secretion of protons to neutralize the alkalinity (Evans et al., 2002; Love, 2001). Issues during the application and removal of CH limit the ability to increase the concentration of CH for effective eradication of *E. faecalis* in the root canal (Estrela et al., 1999; Fava and Saunders, 1999). In particular, extrusion beyond the apical foramen during endodontic treatment should be avoided due to potential tissue irritation, nerve damage, and delayed healing (Gomes et al., 2003; Orucoglu and Cobankara, 2008). Therefore, the development of alternative safe and effective intracanal medicaments is desirable.

Probiotics are living microorganisms offering beneficial effects on host health when administered appropriately (Hill et al., 2014). Family *Lactobacillaceae* as representative probiotics, balance the gut microbiota and enhance gastrointestinal health (Westfall et al., 2017). Conversely, the possibility of bacteremia due to the ingestion of *Lactobacillaceae* by immunocompromised patients has been reported (Salminen et al., 2004). Instead, postbiotics, which are inert microorganisms and/or their components, can be introduced (Vinderola et al., 2022). Postbiotics containing various bioactive components such as lipoteichoic acid (LTA), peptidoglycan, polysaccharides, and proteins have antimicrobial/anti-inflammatory properties and have been used to treat biofilm-related chronic dental diseases including dental caries and chronic periodontitis (Khani et al., 2022; Lee et al., 2018; Twetman and Keller, 2012). In particular, LTA has been shown to effectively inhibit *Streptococcus mutans* biofilm formation and its related diseases (Ahn et al., 2018a, b; Chaudhari and Dwivedi, 2022; Schneewind and Missiakas, 2014). *Lactobacillaceae* LTA can also suppress biofilms of endodontic pathogens, especially those formed by *E. faecalis* (Jung et al., 2019; Kim et al., 2020a).

Although LTA of family *Lactobacillaceae* is a potential endodontic medicament with anti-biofilm effects, it has not been examined as an intracanal medicament in the human tooth root canal model. Therefore, in this study, we examined the effect of LTA derived from *Lacticaseibacillus rhamnosus* GG (Lgg.LTA) as an endodontic medicament in human root canals infected with *E. faecalis*.

## Materials and Methods

### *Bacteria* and Reagents

*E. faecalis* KCTC 3206 and *Lacticaseibacillus casei* KCTC 3260 were obtained from the Korean Collection for Type Cultures. *Lactobacillus acidophilus* KACC 12419 and *L. rhamnosus* GG ATCC 53103 were purchased from the Korean Agricultural Culture Collection and the American Type Culture Collection, respectively. Sterile distilled water (SDW) and CleaniCal^®^ were obtained from Dai Han Pharm and Maruchi, respectively. *N*-methyl-2-pyrrolidone (NMP) and all other reagents, unless otherwise indicated, were purchased from Sigma-Aldrich.

### LTA Purification

Structurally-intact and highly-pure LTA was purified from *L. rhamnosus* GG*, L. casei,* and *L. acidophilus* by butanol extraction, hydrophobic interaction chromatography, and ion exchange chromatography as previously described (Jung et al., 2019). LTAs were lyophilized and quantitated by measuring their dry weight.

### Biofilm Crystal Violet Assays

*E. faecalis* [1 × 10^5^ colony-forming units (CFU)/ml] was grown in the presence or absence of *Lactobacillaceae* LTAs (30 μg/ml) in 96-well plates (Corning) at 37 °C for 5 h under aerobic conditions. After washing with phosphate-buffered saline (PBS) and staining with 1% crystal violet, the wells were rinsed again with PBS. Subsequently, the stained biofilms were dissolved in a dissociation buffer (95% ethanol and 0.1% acetic acid in water), and optical density at 600 nm was determined using a microplate reader (SPARK, Molecular Devices).

### Preparation of Human Dentinal Cylinders

**Fig. 1:**
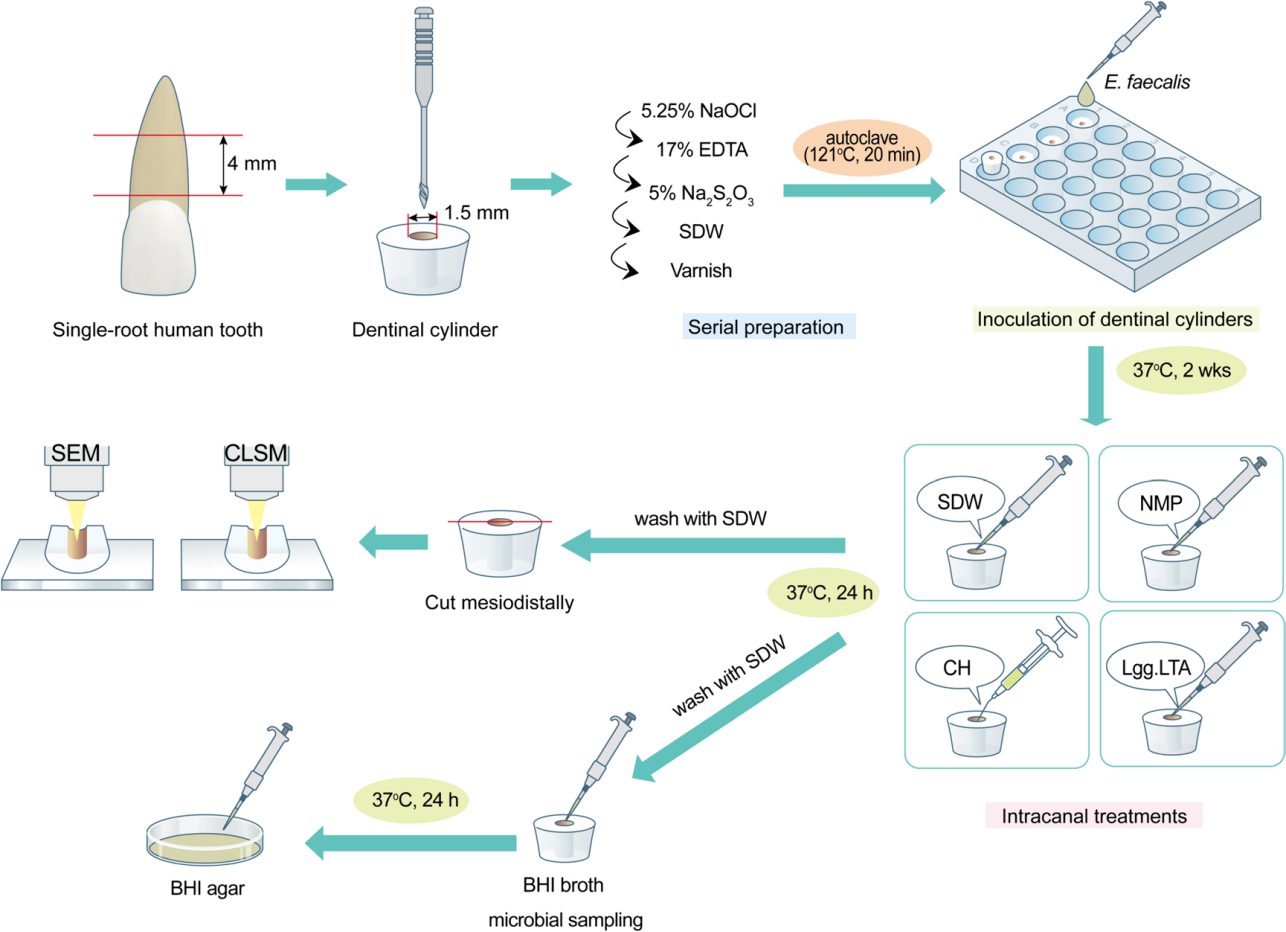
A schematic diagram illustrating the experimental setup. *E. faecalis* was inoculated and cultured in prepared human dentinal cylinders, followed by the application of intracanal medicaments and their subsequent assessments

This study was approved by the Institutional Review Board of Seoul National University Bundang Hospital (B-2309-854-301). Twenty eight human dentinal cylinders were prepared from mature permanent human teeth (upper/lower anterior teeth or premolars with single-root canals) that were extracted for orthodontic or periodontal reasons. To minimize the variability in root canal morphology, we selected only single-rooted teeth with a single canal. Teeth were selected based on visual inspection and radiographs to ensure root integrity without root crack, caries, or resorption. After disinfection in 0.1% thymol for 48 h, the extracted teeth were soaked in SDW before use. Four-millimeter-length dentinal cylinders with a 1.5 mm-diameter root canal were prepared using a method previously described (Bukhari et al., 2018; Ma et al., 2011). The dentinal cylinders were immersed in an ultrasonic bath containing 5.25% sodium hypochlorite (NaOCl) for 4 min, followed by 17% ethylenediaminetetraacetic acid for 4 min to remove the smear layer. To neutralize the effect of NaOCl, a final rinse was performed with 5% sodium thiosulfate in an ultrasonic bath for 4 min. Subsequently, the teeth were rinsed in SDW using an ultrasonic bath for 10 min. Next, nail varnish was applied twice over the entire root surface, followed by autoclaving at 121 °C for 20 min to ensure sterilization. For sterility assessment, two teeth were incubated in Brain Heart Infusion (BHI) broth (BD Bioscience) at 37 °C for two weeks. Afterward, the culture medium was spotted onto BHI agar plates to confirm sterility, and the interior of the root canal was examined using scanning electron microscopy (SEM) (Fig. 1).

### Cultivation of *E. faecalis*

The prepared dentinal cylinders were individually placed in separate wells of a 24-well plate and infected with *E. faecalis* at a concentration of 1 × 10^8^ CFU/ml in BHI broth. Cultures were maintained at 37 °C for two weeks with additional fresh BHI broth added every 3 days. Two teeth specimens were analyzed via SEM to confirm bacterial biofilm formation.

### Intracanal Treatments

Dentinal cylinders inoculated with *E. faecalis* were transferred to 24-well plates. A temporary light-cured resin, Quicks^®^ (DentKist Inc.) was applied to the underside of the tooth specimen, which was used to secure and occlude one end of the root canal. Subsequently, six teeth were allocated to each of the four groups receiving the following intracanal treatments: SDW, 20% NMP (vehicle control) at the minimum bactericidal concentration (MBC) according to previous studies (Kim et al., 2020b, 2022), CH (CleaniCal^®^ containing 30% CH), and Lgg.LTA at 50 μg/ml. After sealing the canal entrance with sterile cotton and Quicks^®^, each well was then filled with 300 μl BHI broth to prevent the external root surface from drying. All dentinal cylinders were incubated for 24 h at 37 °C. The next day, after flushing each root canal with SDW and drying with a sterilized #80 paper point (Diadent Group International Inc.), the dentinal cylinders were used for further assessment.

### Scanning Electron Microscopy

To visually assess the extent of biofilm inside the root canal, SEM imaging was performed. Each dentinal cylinder was split mesiodistally using a No. 15 blade and mallet to expose the root canal, then gently washed with PBS to remove planktonic bacteria. The root sections prefixed with 2.5% glutaraldehyde and 2% paraformaldehyde were dehydrated using ethanol, dried at 37 °C for 24 h, and affixed to mounts. A 20 nm layer of gold–palladium was applied for SEM assessment using an Apreo 2 microscope (Thermo Fisher Scientific) as previously described (Seal et al., 2023). Images were captured at three random areas at 10,000×, 30,000×, and 50,000× magnification at 10.00 kV.

### Confocal Laser Scanning Microscopy

Confocal laser scanning microscope (CLSM) imaging was performed to distinguish between live and dead bacteria, quantitatively analyze biofilm characteristics, and accurately observe biofilms formed deep within dentinal tubules. Each dentinal cylinder was split mesiodistally as aforementioned and then gently washed with PBS to remove planktonic bacteria. The biofilm was stained with 3 ml of LIVE/DEAD^™^ BacLight^™^ Bacterial Viability Kit (Invitrogen) containing SYTO9 and propidium iodide for 5 min. After final washing with PBS, images were randomly observed at three different areas using a CLSM (LSM 800; Carl Zeiss) with simultaneous dual-channel imaging to display red fluorescence (propidium iodide at 490/635 nm) and green fluorescence (SYTO9 at 480/500 nm). The intensity of live and dead cells was quantified using ZEN 3.4 software (Carl Zeiss).

### Re-cultivation and Enumeration of Bacterial Counts

Four dentinal cylinders from each group were placed in 24-well plates and re-fixed with Quicks^®^ for further incubation. The root canals were refilled with 7.5 μl of fresh BHI broth and resealed with sterile cotton and Quicks^®^. The cylinders were then re-incubated for 24 h at 37 °C. The following day, after removing the sealing material, 10 μl of PBS was divided into two portions of 5 μl each and introduced into the canal. After 10 serial dilutions, the solution was transferred into 990 μl of PBS, resulting in a dilution factor of 10^–2^. Subsequently, it was serially diluted by factors of 10^–3^, 10^–4^, and 10^–5^, and 10 μl of each dilution was spotted on BHI agar plates, which were then incubated for 24 h at 37 °C and CFU were enumerated. The counted CFU were visualized through graphs using GraphPad Prism 6 software (GraphPad Software Inc.).

### Statistical Analysis

For crystal violet assays and CLSM, mean ± standard deviation (S.D.) was calculated from triplicate samples for each group. The re-cultivation data were based on quadruplicate samples for each group. The results were statistically analyzed using the Student’s *t*-test at a significance level of *P* < 0.05.

## Results

### LTAs of Various *Lactobacillaceae *Inhibit *E. faecalis* Biofilm Formation

**Fig. 2:**
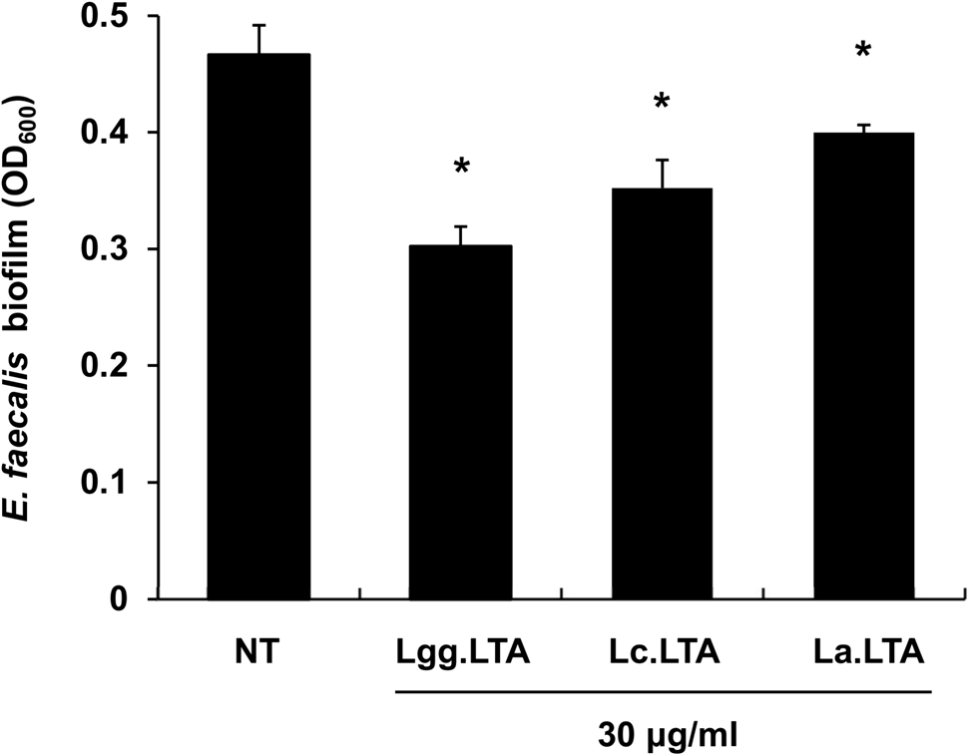
LTAs isolated from various *Lactobacillaceae* species effectively inhibit *E. faecalis* biofilm formation. *E. faecalis* at 1 × 10^5^ CFU/ml was grown to form biofilms in the presence or absence of LTAs (30 μg/ml) purified from *L. rhamnosus* GG*, L. casei,* or *L. acidophilus* in 96-well plates at 37 °C for 5 h under aerobic conditions. Biofilm quantification was performed via crystal violet assay by measuring optical density at 600 nm using a microplate reader. Asterisks (*) indicate significant differences between LTA-treated and non-treated groups (*P* < 0.05). The data shown are the mean values ± S.D. of triplicate samples. NT, Lgg.LTA, Lc.LTA, and La.LTA denotes non-treatment, *L. rhamnosus* GG LTA, *L. casei* LTA, and *L. acidophilus* LTA, respectively

To assess the inhibitory effect of *Lactobacillaceae* LTAs on *E. faecalis* biofilm formation, we initially chose three *Lactobacillaceae* species (*L. rhamnosus* GG, *L. casei*, and *L. acidophilus*) and purified their LTAs. Since *Lactiplantibacillus plantarum* LTA (Lp.LTA) was found to optimally inhibit *E. faecalis* biofilm at a concentration of 30 µg/ml (Jung et al., 2019), this concentration was chosen to compare their anti-biofilm activities against *E. faecalis*. All the aforementioned LTAs resulted in a significant reduction in biofilm quantity compared to the non-treatment control (Fig. 2). Among the LTAs tested, Lgg.LTA exhibited the most effective reduction in biofilm formation. Thus, Lgg.LTA was chosen for the following experiments.

### Lgg.LTA Exhibits Anti-Biofilm Effects in Human Tooth Root Canals

**Fig. 3:**
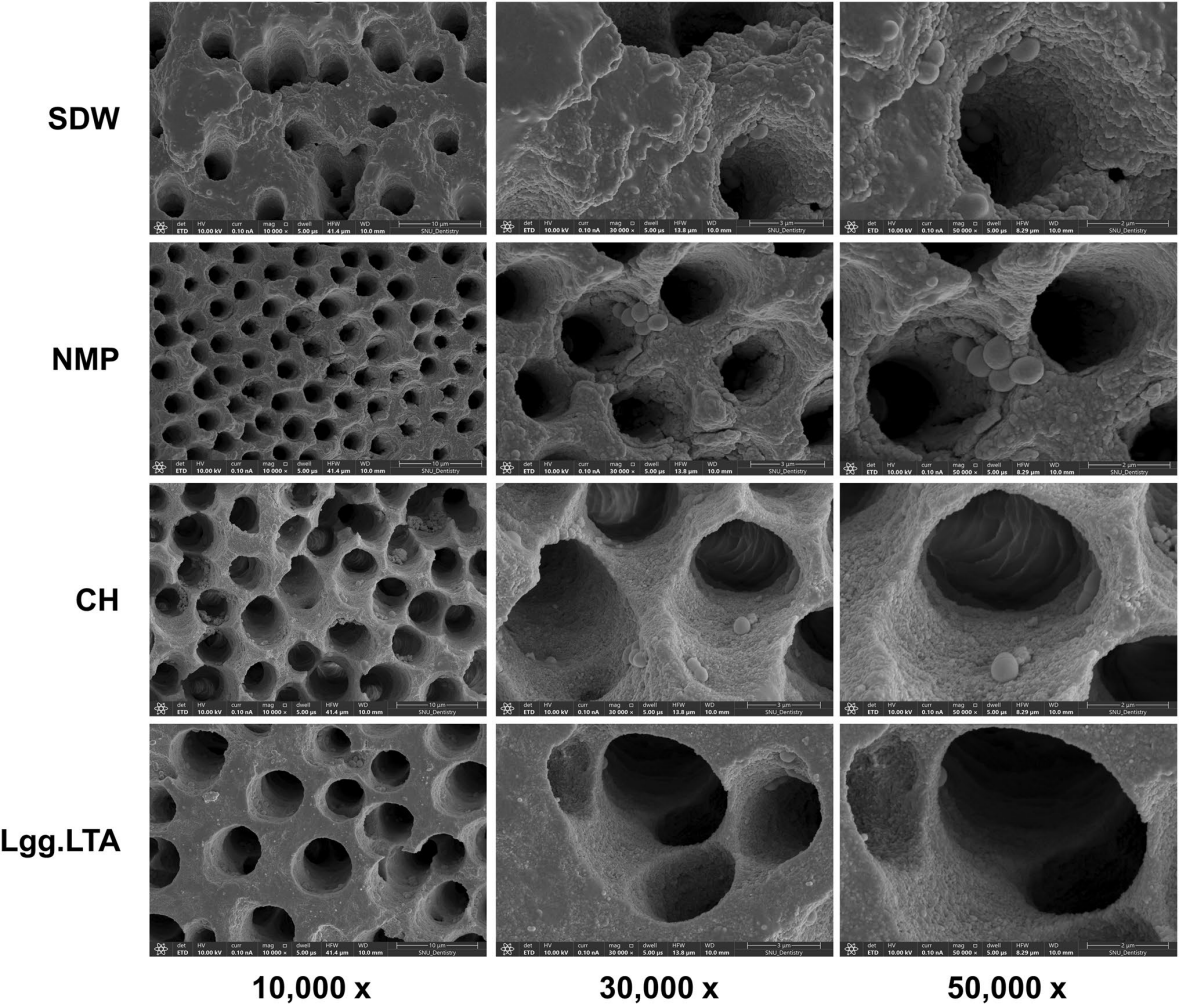
Lgg.LTA exhibits an anti-biofilm effect in the root canal of human teeth. Four millimeter-length dentinal cylinders with a 1.5 mm-diameter root canal were prepared from extracted human teeth. After two-week cultivation of *E. faecalis* (1 × 10^8^ CFU/ml), SDW, NMP, CH, and Lgg.LTA were applied into the root canal for 24 h. After washing with SDW, the root canal was mesiodistally split in half and subjected to SEM imaging. Images were captured at three random areas and representative SEM images of each group are displayed at 10,000×, 30,000×, and 50,000× magnification

Next, we evaluated the potential of Lgg.LTA as an intracanal medicament. Human dentinal cylinders were prepared and infected with *E. faecalis* followed by treatment with Lgg.LTA at 50 µg/ml according to our previous study using human dentinal samples (Kim et al., 2019) or the controls including SDW (negative control), NMP (vehicle control), and 30% CH (positive control). Then, the inner surface of halved specimens from each group were examined using SEM. Representative images show extensive bacterial aggregates covering dentinal tubules in the SDW and NMP groups (Fig. 3). While the NMP group showed a reduced amount of biofilm production compared to the SDW group, it was still higher than in both the CH and Lgg.LTA groups. The CH and Lgg.LTA groups exhibited relatively cleaner dentinal tubules with less biofilm accumulation and bacterial presence. These results suggest that the suppressive effect of Lgg.LTA on *E. faecalis* biofilms is observed in the human tooth model.

### Lgg.LTA Reduces Both Live and Dead Bacteria Within Dentinal Tubules

**Fig. 4:**
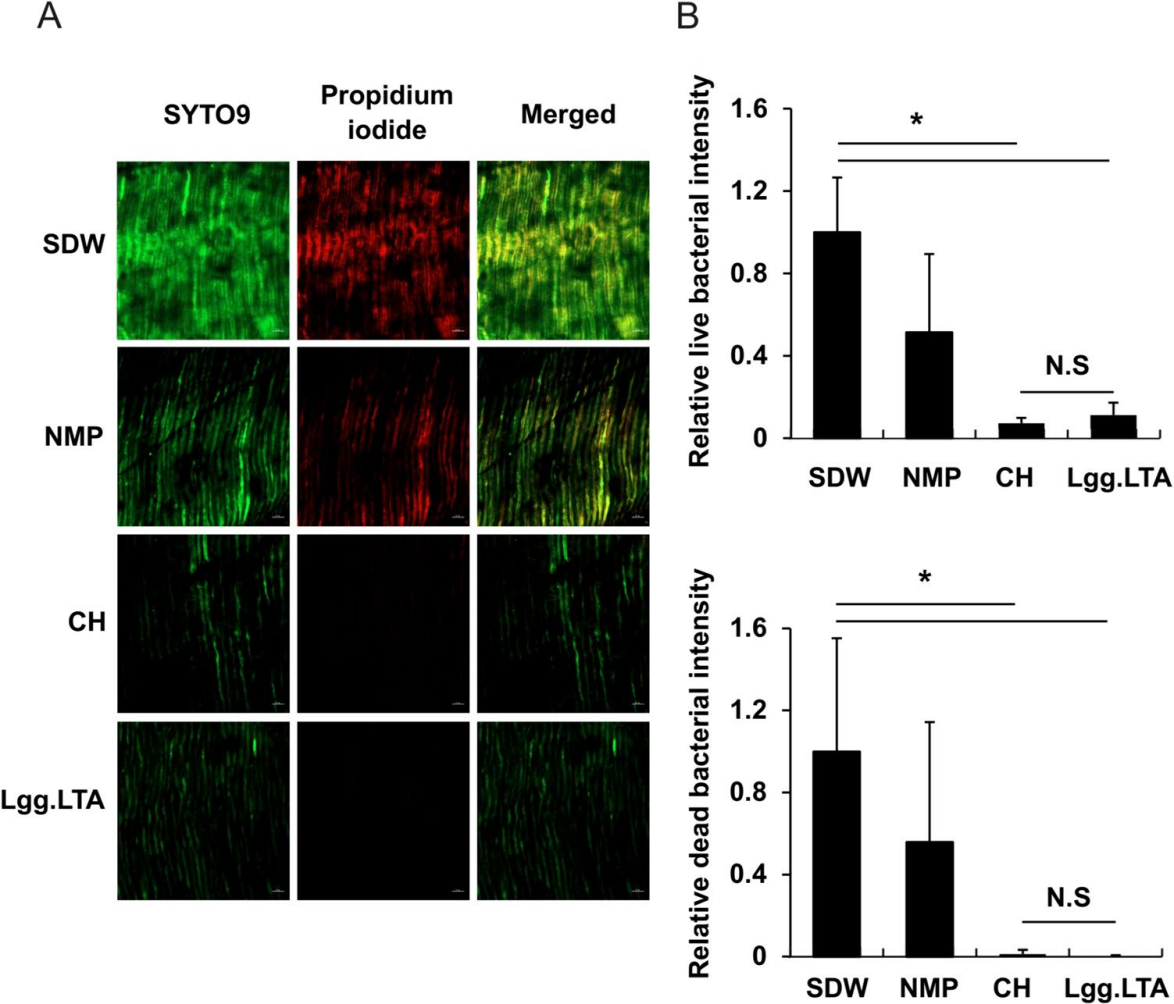
Lgg.LTA reduces the quantity of both live and dead bacteria in the root canal of human teeth (**A**, **B**). The root canals of dentinal cylinders were infected with *E. faecalis* for two weeks followed by intracanal treatment with SDW, NMP, CH, or Lgg.LTA for an additional 24 h. Then, the dentinal cylinders were washed with SDW, mesiodistally halved, stained with SYTO9 (for live bacteria) and propidium iodide (for dead bacteria) using the LIVE/DEAD^™^ *Bac*Light^™^ Bacterial Viability Kit, and subjected to CLSM imaging. **A** Representative images (SYTO9, propidium iodide, and merged) from three similar results are shown. **B** The fluorescence intensity (green and red for live and dead bacteria, respectively) was quantified with ZEN software and graphically presented. Data are mean values ± S.D. The asterisks indicate groups with significantly lower intensity than that of the SDW control group (*P* < 0.05)

It is necessary to investigate the influence of Lgg.LTA on bacteria within dentinal tubules. Thus, live (SYTO9, green) and dead (propidium iodide, red) bacteria within the dentinal tubules were visualized and quantified using CLSM images. As shown in Fig. 4A, both green and red coloration were less prominent in the CH and Lgg.LTA groups than in the SDW and NMP groups, indicating a lower number of both viable and non-viable bacteria in CH and Lgg.LTA treatments. This finding is consistent with the quantification of fluorescence intensity at the individual pixel level (Fig. 4B) and the CH and Lgg.LTA groups exhibited significantly lower intensity than the SDW group. In the NMP group, there was a decrease in bacterial intensity compared to SDW; however, there was no statistical significance. Furthermore, no significant difference was observed between the CH and Lgg.LTA groups in terms of both live or dead cell counts. These findings indicate that Lgg.LTA diminishes the total count of both live and dead bacteria present within the dentinal tubules.

### Lgg.LTA Reduces Live Bacteria in Dentinal Tubules, Implying Minimal Intracanal Reinfection

**Fig. 5:**
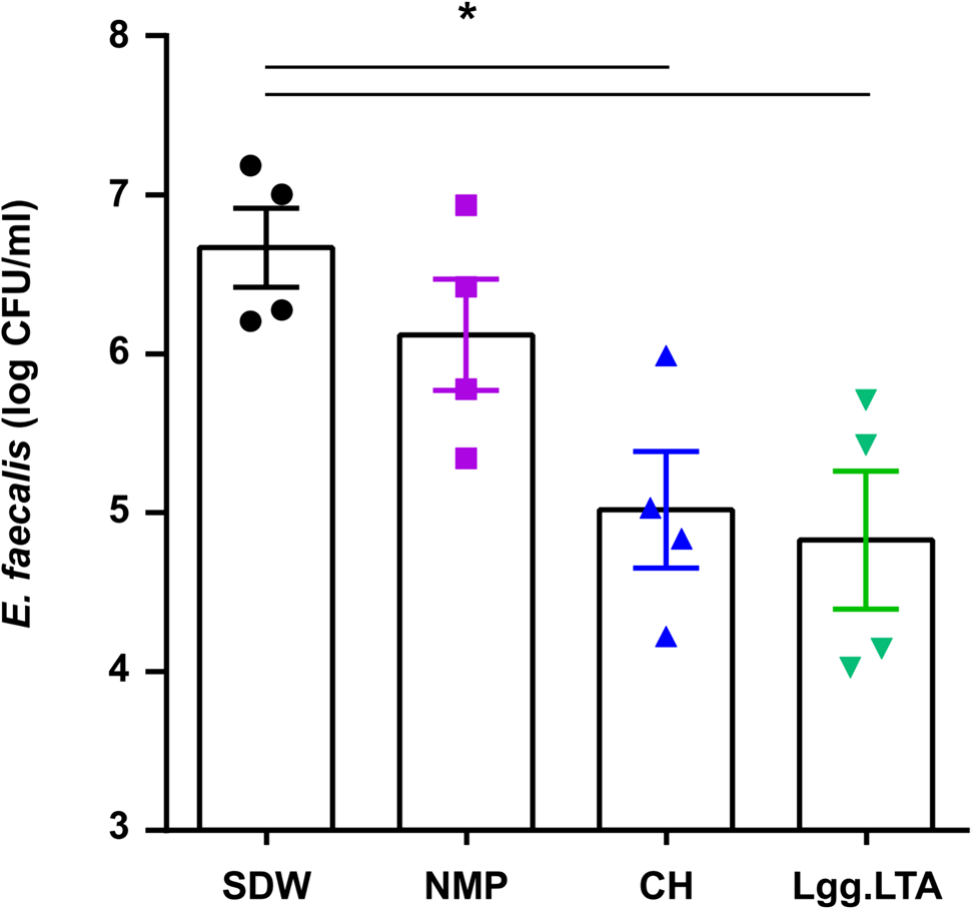
Lgg.LTA reduces bacterial counts within dentinal tubules. After 24 h of intracanal treatment, four dentinal cylinders of each group were transferred to a new plate and BHI was introduced into the root canal. After 24 h, the culture media were serially diluted, inoculated onto BHI agar plates, cultured for additional 24 h, and CFU were enumerated. The mean log CFU/ml ± S.D. was then calculated for each group and compared to the SDW control group. The counted CFU were visualized through graphs using GraphPad Prism 6 software. The asterisks indicate groups with significantly lower intensity than that of the SDW control group (*P* < 0.05)

We assumed that if less *E. faecalis* biofilm is present within the root canals in SEM images, the degree of recontamination within the root canal would also be low. Therefore, 24 h after applying and rinsing the medicament, sterile BHI broth was added to the root canal. After re-cultivation, both the CH and Lgg.LTA groups showed significantly reduced bacterial counts compared to SDW (Fig. 5). In the NMP group, a decrease in CFU was observed, but there was no significant difference between the NMP and SDW groups. Applying Lgg.LTA as an intracanal medicament can decrease bacterial counts within dentinal tubules, resulting in less possibility of re-contamination in the root canals after Lgg.LTA treatment.

## Discussion

We investigated whether Lgg.LTA, as demonstrated on plates (Jung et al., 2019) and dentin slices (Kim et al., 2019), could yield similar anti-biofilm effects in human teeth with dentinal tubules. This study proved that the intracanal application of Lgg.LTA has an anti-biofilm effect in *E. faecalis*-infected root canals. Additionally, fewer bacterial cells were observed in the dentinal tubules of Lgg.LTA-treated root canals. Collectively, Lgg.LTA could be used as an effective intracanal medicament, resulting in significantly cleaner dentinal tubules with reduced bacterial presence and biofilm formation.

Lgg.LTA effectively inhibits *E. faecalis* biofilm formation in dentinal cylinders from human extracted teeth as well as on inorganic surfaces. Other studies have also demonstrated the anti-biofilm effects of various LTAs on *E. faecalis*. LTAs derived from *L. plantarum*, *L. casei*, *L. rhamnosus*, and *L. acidophilus* can individually suppress and disrupt *E. faecalis* biofilm formation on plates and human dentin slices (Jung et al., 2019). Furthermore, LTAs are not limited to inhibiting *E. faecalis* biofilms alone. For instance, Lp.LTA has been shown to inhibit multispecies oral biofilms composed of *Actinomyces naeslundii, Ligilactobacillus salivarius, S. mutans,* and *E. faecalis* (Kim et al., 2019). Similarly, Lp.LTA suppressed biofilm formation of *S. mutans* and also *Staphylococcus aureus* (Ahn et al., 2018a, b).

While Lgg.LTA and CH exhibited similar inhibitory effects on biofilms, their action mechanisms seem to differ. CH exerts a direct bactericidal effect with hydroxyl ions, whereas LTA does not possess any bactericidal properties but interferes with biofilm formation and even disrupts the preformed biofilm (Ahn et al., 2018a, b; Jung et al., 2019; Siqueira and Lopes, 1999). A previous study on *S. aureus* biofilms suggests that Lp.LTA enhances autoinducer-2 (AI-2), a quorum sensing molecule, consequently inhibiting the expression of genes for exopolysaccharide production, thus suppressing biofilm formation (Ahn et al., 2018a, b). The AI-2-dependent biofilm regulation mechanism is also present in *E. faecalis.* In a *luxS* mutant strain of *E. faecalis*, the overexpression of genes related to biofilm formation, such as *esp* and *ace*, was observed (He et al., 2016). Therefore, further research is needed to investigate whether Lgg.LTA affects the expression of the *luxS* gene to regulate AI-2 together with *esp* and *ace* genes. Besides, it is also possible that LTA stimulates bacteria to produce digestive enzymes to disrupt the preformed biofilm.

An appropriate vehicle for endodontic medicaments should also be considered to effectively deliver LTA into the tooth root canal. NMP is recently being considered as a carrier for CH in clinical settings because it has higher solubility compared to propylene glycol, a commonly used CH carrier, making it easier to remove (Lim et al., 2017). Therefore, NMP was chosen as the carrier in this study. NMP, which is also a component of CleaniCal^®^, serves as an aqueous solvent with the ability to dissolve various organic and inorganic compounds (Kim et al., 2020b, 2022). Its reduced surface tension has been acknowledged to enhance the penetration of drugs (Sanghvi et al., 2008). In addition, NMP itself possesses the ability to disrupt microbial biofilms (Kim et al., 2022). The current study also indicates that NMP alone can reduce bacterial biofilms, though its effectiveness appears to be lower than CH or Lgg.LTA. Remarkably, NMP, known as a biocompatible material, is not supposed to alter LTA structure and/or its anti-biofilm action. Further study is needed to assess other delivery carriers such as propylene glycol and methylcellulose.

The significance of this study lies in confirming that Lgg.LTA effectively inhibits *E. faecalis* biofilm production in an extracted tooth model, suggesting the potential of LTAs to treat endodontic infection. Of note, we previously reported that Lp.LTA exhibits a minimal immunostimulatory potential, unlike LTAs from pathogenic bacteria such as *S. aureus* (Ryu et al., 2009). Additionally, Lp.LTA showed anti-inflammatory activities in human intestinal epithelial cells by inhibiting the expression of IL-8 induced by Pam2CSK4 or poly I: C (Kim et al., 2017; Noh et al., 2015). Concordantly, according to a recent study assessing the cytotoxicity of LTAs from various *Lactobacillaceae* strains (*Limosilactobacillus reuteri*, *L. acidophilus*, and *L. plantarum*), no adverse effects were observed in a mouse macrophage cell line even when the concentration of LTA was increased to 100 μg/ml. Moreover, those LTAs decreased the expression of proinflammatory cytokines TNF-α and IL-6 while increasing the expression of the anti-inflammatory cytokine IL-10 (Lu et al., 2022). Therefore, similar investigations are necessary to verify the safety of Lgg.LTA, particularly when targeting human periodontal ligament cells or alveolar bone cells. Evaluating the response of host cells to Lgg.LTA will provide valuable insight into the potential application of LTAs in treating apical periodontitis. Furthermore, additional ex vivo studies are necessary to determine the optimal dosage, duration, and carrier for the application of LTA from a specific *Lactobacillaceae* strain, specifically targeting clinical strains extracted from patients with actual apical periodontitis.

Within its limitations as an *in-vitro* and *ex-vivo* study rather than *in-vivo* clinical studies with actual human oral cavities, this study is the first to demonstrate the potential for using Lgg.LTA as an intracanal medicament in actual tooth models. However, the real root canal system, with its immune and nerve components, may be more complex and exhibit greater variability than the *ex-vivo* tooth model. Therefore, further studies are needed to evaluate the effects of Lgg.LTA in actual human root canals or through animal experiments to substantiate its efficacy.

## Data Availability

All data are available upon any reasonable request via the corresponding author.
